# Identification of Differentially Methylated CpG Sites in Fibroblasts from Keloid Scars

**DOI:** 10.3390/biomedicines8070181

**Published:** 2020-06-28

**Authors:** Mansour A. Alghamdi, Hilary J. Wallace, Phillip E. Melton, Eric K. Moses, Andrew Stevenson, Laith N. Al-Eitan, Suzanne Rea, Janine M. Duke, Patricia L. Danielsen, Cecilia M. Prêle, Fiona M. Wood, Mark W. Fear

**Affiliations:** 1 Department of Anatomy, College of Medicine, King Khalid University, Abha 61421, Saudi Arabia; m.alghamdi@kku.edu.sa; 2 Genomics and Personalized Medicine Unit, College of Medicine, King Khalid University, Abha 61421, Saudi Arabia; 3 School of Medicine, The University of Notre Dame Australia, Fremantle 6959, Australia; hilary.wallace@nd.edu.au; 4 Burn Injury Research Unit, School of Biomedical Sciences, Faculty of Health and Medical Sciences, The University of Western Australia, Perth 6009, Australia; andrew@fionawoodfoundation.com (A.S.); janine.duke@uwa.edu.au (J.M.D.); fiona.wood@health.wa.gov.au (F.M.W.); 5 Centre for Genetic Origins of Health and Disease, Faculty of Health and Medical Sciences, The University of Western Australia, Perth 6009, Australia; phillip.melton@uwa.edu.au (P.E.M.); eric.moses@uwa.edu.au (E.K.M.); 6 School of Pharmacy and Biomedical Sciences, Faculty of Health Science, Curtin University, Perth 6102, Australia; 7 Department of Applied Biological Sciences, Jordan University of Science and Technology, Irbid 22110, Jordan; lneitan@just.edu.jo; 8 Department of Biotechnology and Genetic Engineering, Jordan University of Science and Technology, Irbid 22110, Jordan; 9 Burns Service of Western Australia, Perth Children’s Hospital and Fiona Stanley Hospital, Department of Health, Perth 6009, Australia; Suzanne.Rea@health.wa.gov.au; 10 Department of Dermatology and Copenhagen Wound Healing Center, Copenhagen University Hospital, DK-2400 Copenhagen NV, Denmark; patriciadanielsen@yahoo.dk; 11 Institute for Respiratory Health, Faculty of Health and Medical Sciences, The University of Western Australia, Perth 6009, Australia; cecilia.prele@uwa.edu.au; 12 Fiona Wood Foundation, Perth 6150, Australia

**Keywords:** keloid scars, DNA methylation, wound healing, epigenetics

## Abstract

As a part of an abnormal healing process of dermal injuries and irritation, keloid scars arise on the skin as benign fibroproliferative tumors. Although the etiology of keloid scarring remains unsettled, considerable recent evidence suggested that keloidogenesis may be driven by epigenetic changes, particularly, DNA methylation. Therefore, genome-wide scanning of methylated cytosine-phosphoguanine (CpG) sites in extracted DNA from 12 keloid scar fibroblasts (KF) and 12 control skin fibroblasts (CF) (six normal skin fibroblasts and six normotrophic fibroblasts) was conducted using the Illumina Human Methylation 450K BeadChip in two replicates for each sample. Comparing KF and CF used a Linear Models for Microarray Data (Limma) model revealed 100,000 differentially methylated (DM) CpG sites, 20,695 of which were found to be hypomethylated and 79,305 were hypermethylated. The top DM CpG sites were associated with *TNKS2*, *FAM45B*, *LOC723972*, *GAS7*, *RHBDD2* and *CAMKK1*. Subsequently, the most functionally enriched genes with the top 100 DM CpG sites were significantly (*p* ≤ 0.05) associated with SH2 domain binding, regulation of transcription, DNA-templated, nucleus, positive regulation of protein targeting to mitochondrion, nucleoplasm, Swr1 complex, histone exchange, and cellular response to organic substance. In addition, *NLK, CAMKK1, LPAR2, CASP1*, and *NHS* showed to be the most common regulators in the signaling network analysis. Taken together, these findings shed light on the methylation status of keloids that could be implicated in the underlying mechanism of keloid scars formation and remission.

## 1. Introduction

Wound healing in the human body is one of the most complex and progressive processes that require the involvement of several different molecular and cellular events [1,2]. The normal healing process involves multi-dynamic stages in three overlapping phases: the inflammation reaction, cellular elements proliferation and synthesis, and remodeling [2,3]. The latter phase outcomes are clinically predominant where collagen deposition occurs [4]. When the healing process is adequate, inconspicuous harmless scars, the normotrophic, are formed [5].

In response to aberrant healing of skin injuries and irritation, undesirable scars are raised [6,7]. Scars can fall into both hypertrophic and keloids that are not necessarily the same, but can be difficult to differentiate [4,8]. Keloids are often distinguished by their growing to the surrounding healthy skin beyond the margins of the original tissue lesion [8,9]. Keloid scars are benign dermal fibrotic tumors clinically characterized by the excessive production and deposition of the extracellular matrix (ECM) components [4,10,11]. Although the pathogenesis of keloids is poorly elucidated, some genetic and environmental factors, as well as epigenetic mechanisms, have been involved, resulting in dysregulation of the tissue repair and regeneration processes [3,12,13]. 

Interests have recently progressed toward the field of cutaneous epigenetics as a vital mechanism in regulating gene expression. DNA methylation is one of the most potent epigenetic changes that is common in cytosine-phosphoguanine (CpG) dinucleotides in which cytosine residue is bind to a methyl group (CH3) at position C5 [12,14]. Methylation of the DNA is essential during early normal development and contributes to the natural phenotypic variation in humans [15,16]. The aberrant addition (hypermethylation), or removal (hypomethylation) of the methyl group can either decrease or increase the rate of gene expression [17,18]. Epigenetic modification, including DNA methylation, has been observed in several human disease, including keloid scars [18,19,20,21,22]. These findings strongly suggested that DNA methylation is crucial in maintaining genome stability and plays an important role in disease pathogenesis. Most DNA methylation is known to occur at CpG sites [23]. 

These studies mainly focused on CpG islands methylation, which are regions with high cluster of CpG sites. Therefore, this study was undertaken to investigate the methylation levels of CpG sites in all genomic regions in keloid fibroblasts compared to the normal skin and normotrophic scar fibroblasts to further understand the role of DNA methylation as an epigenetic modification in keloid pathogenesis.

## 2. Experimental Section

### 2.1. Subjects

Keloid tissue samples were obtained from 12 patients (8 males and 4 females with an average age of 34 years) (Table 1) who were enrolled in a clinical trial which included excision of a keloid scar. These subjects had received no previous treatment of the keloid scar within 6 months of surgical excision. A full medical history was taken prior to excision and clinical examination was performed by a surgeon to confirm the diagnosis of keloid scar. The causes of keloid scar include immunization site, surgery, non-burn trauma, acne, and burn injury. Control samples (*n* = 12) were normotrophic burn scar tissue (Vancouver Scar Scale: height sub-score = 0) and matched normal skin collected from 6 male subjects with an average age of 24.5 years who had sustained a previous unilateral burn injury (Table 1). The clinical criteria used to differentiate a keloid scar from a normotrophic scar include a history of continuous growth outside the boundaries of the original wound and symptoms such as pain and itch. 

The study was performed in accordance with the relevant National Health and Medical Research Council (NHMRC) of Australia’s ethical statements and guidelines. Ethical approval for all tissue collection was obtained from the Human Research Ethics Committees of the University of Western Australia (RA/4/1/5604; Date: 03/09/2012) and Royal Perth Hospital (EC2009/114 and EC 2012/067; Date: 03/09/2012). All participants provided written informed consent. 

### 2.2. Isolation and Culture of Fibroblasts from Keloid Scar and Control Samples

Fibroblasts were isolated from fresh tissue by the explant method. This method was slightly modified from previously published methods [24,25]. Cell growth was maintained until the second passage (P2) and then cells were frozen in liquid nitrogen and stored until further experiments. 

### 2.3. DNA Extraction and Bisulfate Conversion

DNA was extracted from the fibroblasts (P2) using a QIAamp DNA Mini kit (Cat. No. 51304, Qiagen, Hulsterweg, The Netherlands) and Promega Wizard SV Genomic DNA system (Cat. No. A2360, Promega, Madison, WI, USA) as per the manufacturers’ instructions. The quality and quantity of extracted DNA were measured using a NanoDrop-2000 spectrophotometer (Thermo Fisher Scientific, Waltham, MA, USA). Bisulfite conversion of 800 ng of DNA was carried out on 24 samples using the EZ DNA Methylation kit (Cat. No. D5001, Zymo Research, Irvine, CA, USA) according to the manufacturer’s instructions. 

### 2.4. DNA Methylation Assay

A minimum of 500 ng genomic DNA was amplified, fragmented, and hybridized onto the Illumina Human Methylation 450K BeadChip (Cat. No. WG-314-1003, Illumina, San Diego, CA, USA) according to the manufacturer’s protocol (Illumina 2013). This BeadChip processes 12 samples per array. Two replicates of the methylation assay were performed per sample. The first BeadChip included bisulfite-converted DNA from 6 normal skin fibroblasts and 6 normotrophic fibroblasts. The second BeadChip included bisulfite-converted DNA from 12 keloid scar fibroblasts.

### 2.5. Data Processing for the 450k Methylation Array

A computational R package (RnBeads) was adapted to process and analyze the raw intensity data from methylation chip (IDAT files) [26]. The dataset was subjected to filtering procedures, background subtraction, and normalization. The filtering stages included the removal of sites overlapped with SNPs, greedycut algorithm, the removal of probes with specific contexts and missing values, and the removal of probes with beta values exhibiting standard deviation lower than 0.005. The methylumi package (method “noob”) was used for background subtraction and the Beta-Mixture Quantile Normalization (BMIQ) method was used to normalize the methylation beta values. As keloid fibroblasts and control fibroblasts were run on separate BeadChips, a batch effect may occur. RnBeads uses the Surrogate Variable Analysis (SVA) package to visualize and adjust for batch effects during differential methylation analysis. As a final outcome, a summary of the changes at CpG sites was generated.

### 2.6. Differential Methylation Statistical Analysis

Differential methylation (DM) analysis comparing keloid fibroblast (KF) DNA with control fibroblasts (CF) DNA was carried out at the CpG site. The comparison was computed using a Linear Models for Microarray Data (Limma) method [27], which has been adapted by the RnBeads package for use in methylation arrays. The linear models were employed and fitted using an empirical Bayes approach on derived M-values. The Benjamini and Hochberg (B-H) 5% false discovery rate (FDR) was used to correct for multiple testing. The DM for each CpG sites was computed based on three measures: the beta difference in methylation means between KF and CF, the log2 of the quotient in methylation, and the differential methylation *p*-value using Limma. Using these three measures, each CpG site was given a rank. The combined rank was computed as the maximum (=worst) rank among the three ranks. A smaller combined rank indicates that the CpG sites exhibit more DM [26]. The top-ranking 100,000 sites with the smallest combined rank score were selected for further analysis.

### 2.7. Enrichment and Pathway Analysis

The Database for Annotation, Visualization, and Integrated Discovery (DAVID) v.6.8 [28,29], was utilized to conduct GO term enrichment analysis of the genes corresponding to the top 100 CpG sites. The GO terms included three criteria: biological process (BP), cellular component (CC), and molecular function (MF). A *p*-value ≤ 0.05 was used as a cut-off threshold. 

### 2.8. Interaction Network Analysis

The interaction network analysis of genes associated with the top 100 DM CpG sites was explored using the Signaling Network Open Resource 2.0 (SIGNOR) [30]. The type of relation was selected to include only “all” interactions with a relaxed layout and score of “0.0”. 

## 3. Results

### 3.1. Samples Clustering 

**Figure 1:**
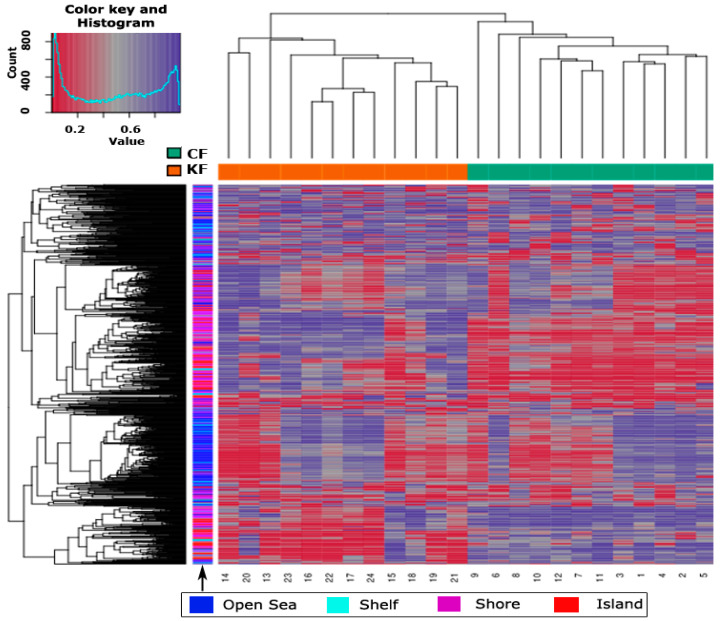
Hierarchal clustering of the top 1000 most variable loci across the 24 samples. Clustering used average linkage and Manhattan distance. The top *x*-axis shows the study groups, where KF and CF stand for keloid fibroblasts and control fibroblasts, respectively. The bottom *x*-axis shows the samples identification numbers, 13–24 represent KF and 1–12 represent CF. The color key and histogram of the heatmap defines the pattern of methylation, values of 0 (red color) and 1 (purple color) indicate decreased and increased methylation, respectively. The distribution of the 1000 most variable loci across the different cytosine-phosphoguanine (CpG) regions (open sea, shelf, shore and island) is shown with color coding on the bottom *x*-axis.

**Figure 2:**
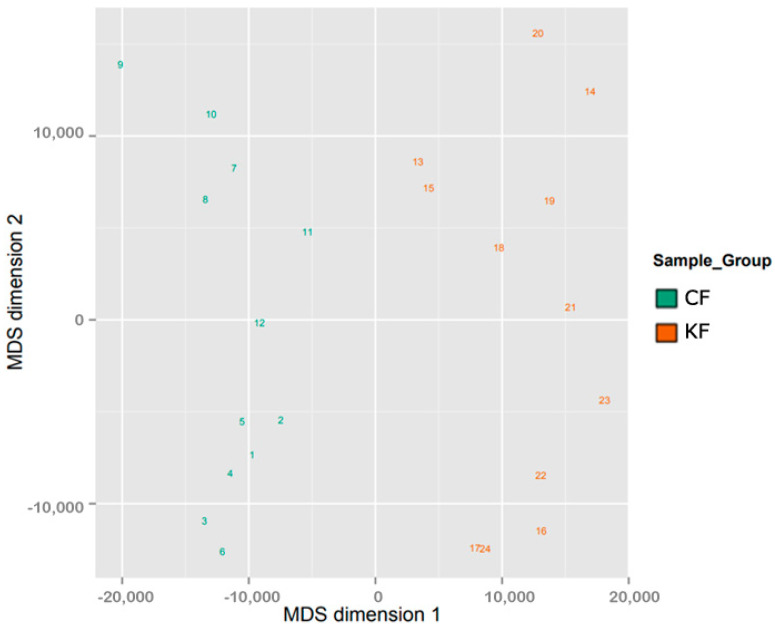
Two Multidimensional scaling (MDS) of the sample groups. The scatter plots show the coordinates of the control fibroblasts (CF) and keloid fibroblasts (KF) samples after performing Kruskal’s multi-dimensional scaling based on the matrix of the average methylation levels and Manhattan distance. The plot of 24 samples (1–12 CF and 13–24 KF) shows that samples cluster according to methylation level, as expected.

The samples sharing similar methylation patterns showed an expected hierarchal clustering based on all the methylation values of the top 1000 most variable loci (Figure 1). In a study conducted by our research group, we have found no statistical significance in methylation level between normotrophic burn scar fibroblasts and matched normal skin fibroblasts (data not published). Hence, fibroblasts from both tissue types (normotrophic burn scar and normal skin) were used in this study as CF to increase statistical power. Figure 1 shows one hierarchal clustering of CF isolated from normotrophic burn scar and normal skin explaining the similarity in methylation patterns and another clustering of KF isolated from keloid scar. The dataset was inspected for a strong signal in the methylation values using a dimension reduction test by implementing multidimensional scaling (MDS) method (Figure 2). MDS confirmed that the difference in methylation level between KF and CF dominates the analysis.

### 3.2. Differential Methylation of CpG Sites

**Figure 3:**
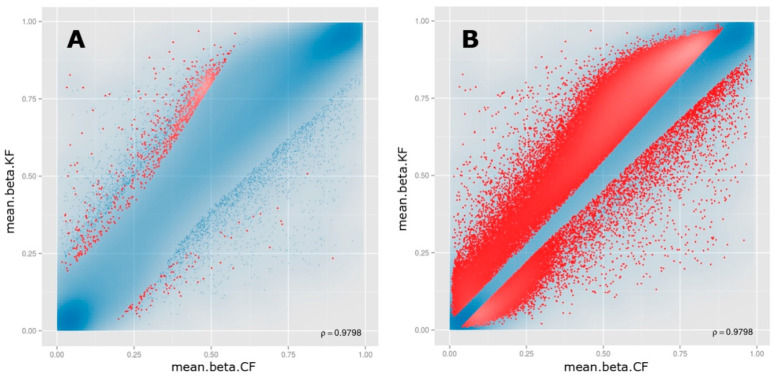
Scatter plots for the (**A**) top-ranking 1000 and (**B**) top-ranking 100,000 differentially methylated CpG sites. For each plot, the mean β values of control fibroblasts (mean.beta.CF) are on the *x*-axis, while the mean β values of keloid fibroblasts (mean.beta.KF) are on the *y*-axis. Methylation levels (β) varied between 0 (unmethylated) and 1 (fully methylated). Blue points represent variable differentially methylated sites.

The top 100,000 sites ranked according to the combined rank score were selected to identify DM CpG sites in KF. Of the 100,000 CpG sites, 20,695 CpG sites were found to be hypomethylated (beta difference range = −0.030 to −0.67) and 79,305 CpG sites were hypermethylated (beta difference range = 0.030 to 0.79) in KF compared to CF with an adjusted *p*-value (FDR) ≤0.11 (Figure 3). The log2 of the quotient in methylation between KF and CF had a minimum value of −3.34 and a maximum value of 4.42. The top 100 DM CpG sites with the lowest combined rank score are shown in Table S1. The beta difference of the list of 100 DM sites ranged from −0.22 to −0.42 for hypomethylated genes and ranged from 0.23 to 0.60 for hypermethylated genes with an adjusted *p*-value (FDR) ≤1.72 × 10^−6^.

### 3.3. Functional Enrichment Analysis

The genes associated with the top 100 DM CpG sites were subject to functional enrichment analyses using the DAVID webtool. The most significant gene ontology (GO) terms (*p*-value ≤ 0.05) are shown in Table 2. On the MF level, the associated genes were mainly enriched for “SH2 domain binding”, on the CC level, genes were enriched for “nucleus”, “nucleoplasm”, and “Swr1 complex”. On the BP level, the associated genes were enriched for four terms including “regulation of transcription, DNA-templated”, “positive regulation of protein targeting to mitochondrion”, “histone exchange”, and “cellular response to organic substance”.

### 3.4. Interaction Network

**Figure 4:**
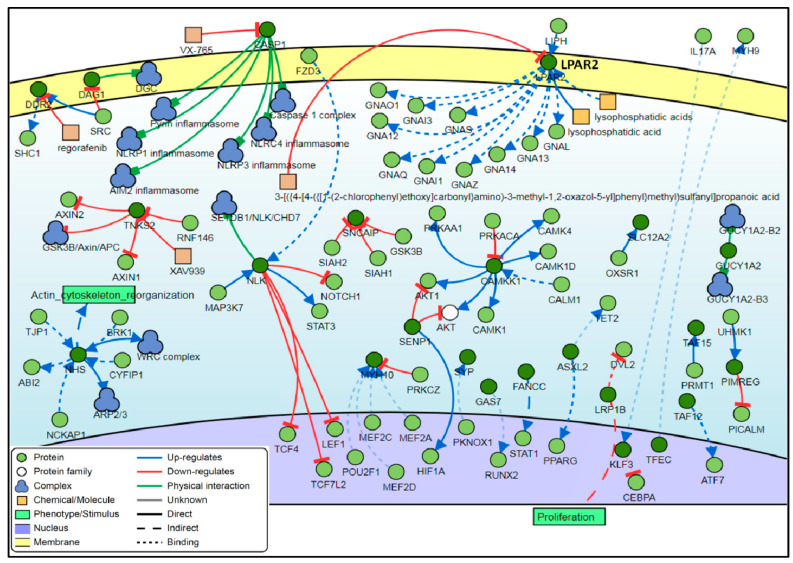
Interaction network of the genes associated with the top-ranking 100 CpG sites. Among these genes, five genes (*NLK*, *CAMKK1*, *LPAR2*, *CASP1* and *NHS*) have the most relationships and interactions with proteins and molecules. The type of interactions, proteins and molecules are shown in the color, coded legend at the right corner of the figure.

Analysis of the interaction between the genes associated with the top 100 DM CpG sites showed that five genes were found to be common regulators with a minimum of 7 connectivities each. These genes are the *NLK*, *CAMKK1*, *LPAR2*, *CASP1* and *NHS* genes (Figure 4).

## 4. Discussion

Keloid scarring is an aggressive skin disease with unclear pathogenesis. It is characterized by excessive fibroblast proliferation and collagen accumulation in the ECM that does not regress over time [31]. Keloid scars are an ongoing clinical challenge with no single effective treatment regimen. Nevertheless, clinicians use several modalities for scar prevention and management, including massage therapy, pressure garments, adhesive tape support/silicone gel sheeting, intralesional corticosteroid injections, laser and light-based therapy, cryotherapy, radiotherapy, 5-fluorouracil, interferon injections, bleomycin injections, and surgery [32]. Among these wide treatment strategies, intralesional steroid injections remain the first-line treatment for many clinicians alone or in combination with other treatment modalities [33]. A widely used intralesional corticosteroid is triamcinolone acetonide [33]. Treatment with triamcinolone alone proved to be more effective than verapamil for the prevention of keloid scar recurrence after surgical excision [34]. Compared to 5-fluorouracil and bleomycin, triamcinolone is more effective in preventing recurrence and reducing scar size and symptoms [33]. However, a combined treatment of triamcinolone with other treatment modalities such as verapamil, 5-fluorouracil, and laser therapy showed better outcomes and patient satisfaction [33]. Depending on the size and location of scars, the recommended concentration of triamcinolone ranges from 10 to 40 mg/mL [35].

Unlike genetic alterations, epigenetic changes are potentially reversable [36]. Therefore, the identification of epigenetic targets could lead to therapeutic interventions. DNA methylation is an epigenetic gene regulatory mechanism that has been found to play a fundamental role in cancer [37], fibrotic disorders [15,38,39], cutaneous diseases [18,22,40,41,42], and wound healing [20,43]. DNA methylation is known to occur commonly at CpG islands and was traditionally linked to transcriptional silencing. These CpG islands are found in high densities in the promoter regions of genes, where transcription of DNA begins, and thus regulate gene transcription [44,45]. This study has explored whether altered collagen deposition by keloid fibroblasts could at least in part be explained by epigenetic changes sustainably and heritably altering the gene expression profile in these cells. Our findings showed significant DNA methylation changes at the CpG sites across multiple genes in KF DNA compared to CF DNA. 

The overgrowth and hyperproliferative nature of keloids are common features with tumorigenesis, in addition to epigenetic and genetic aberration. Of the differentially methylated 100,000 CpG sites in our analysis, more than two-thirds were hypermethylated. Consistent with several cancers-linked DNA hypermethylation, such as in prostate cancer (PCa) [46], and breast cancer [47]. In contrast, more hypomethylation profiles found in keloids genome, frequently in the non-promoter regions [17].

Among the 100 top-ranking DM CpG sites in KF compared to CF, the top seven genes were tankyrase 2 (*TNKS2*), hepatopoietin PCn127 (*LOC723972*), family with sequence similarity 45 member B (*FAM45B*), growth arrest specific 7 (*GAS7*), neuroblastoma breakpoint member 3 (*NBPF3*), rhomboid domain containing 2 (*RHBDD2*), and family with sequence similarity 64 member A (*FAM64A*). *TNKS2* (cg11963436) and *LOC723972* (cg21581312) were reported to be among the 50 DM CpG sites associated with lung function in cystic fibrosis patients [48]. From a methylation-based Epigenome-Wide Association Study (EWAS) in Parkinson’s disease (PD) identifying 9983 DM genes, *FAM45B*, *LOC723972*, and *TNKS2* were among 20 unique DM genes in the patients. In further evaluation, *TNKS2* (cg11963436) was one of two significantly confirmed DM genes that hypermethylated in PD patients [49]. Using the Illumina Human Methylation 450K BeadChip, *LOC723972* (cg21581312) CpG site was hypomethylated in cervical squamous cell carcinoma (SCC) as well as in cervical intraepithelial neoplasia grade 3 (CIN3) compared with the normal tissues [50]. Methylation profiles in colorectal cancer (CRC) showed 50 significantly hypermethylated CpG sites located within six genes in African American patients, including *GAS7* gene [51]. Moreover, among 355 methylated CpG sites in CRC, *GAS7* was one of 59 significantly methylated sites in CRC tissues compared to normal tissues [52]. Furthermore, several epigenomic analyses revealed the hypermethylation pattern of *GAS7* in lung cancer [53,54], prostate cancer [55], CRC [56], and pancreatic endocrine tumors (PETs) [57]. In order to investigate the strong association of the obesity-associated (*FTO*) gene variants and obesity with epigenetic changes, a genome-wide methylation scan in obese and normal weight females identified 20 DM sites in obese females, of which one was hypomethylated site in *NBPF3* gene [58]. There are little or no reports on the methylation status of *RHBDD2*, but it was found to be overexpressed in CRC [59], breast cancer [60,61], and as a pathogenic gene in familial non-medullary thyroid cancer (FNMTC) [62]. The *FAM64A* gene correlated in differentially methylated regions (DMRs) containing multiple CpG sites associated with nevus count (n-DMRs), which is a strong risk factor, contributed to melanoma pathogenesis [63].

An interaction network analysis of the top 100 DM CpG sites showed five common regulator genes: nemo-like kinase (*NLK*), calcium/calmodulin-dependent protein kinase 1 (*CAMKK1*), lysophosphatidic acid receptor 2 (*LPAR2*), caspase 1 (*CASP1*), and Nance-Horan syndrome (*NHS*). The mitogen-activated protein kinase (MAPK) member, *NLK* is involved in a variety of signaling pathways as well as in several types of cancer. *NLK* was found to positively regulate the activation of CCAAT/enhancer binding proteins (C/EBPs) signaling cascade triggered by the proinflammatory cytokine, interleukin-1 (IL-1) [64]. In contrast, it was found to negatively regulate the ternary transcriptional complex in the Notch signaling pathway that is crucial in cell fates of metazoan tissues [65]. Aberrant expression of NLK has been associated with the initiation or progression of oral squamous cell carcinoma [66], laryngeal cancer [67], non-small-cell lung cancer (NSCLC) [68,69], and CRC [70]. The *CAMKK1* gene mediated the upstream activation of AMP-activated protein kinase (AMPK) by phosphorylation of the α-subunit threonine 172 (Thr172) residue. AMPK is a key regulator in various cellular events and targets, such as in the treatment of type 2 diabetes (T2D) and maintaining homeostasis of the cellular energy [71,72]. The expression levels of *LPAR2* have a critical role in carcinogenesis and during tumor progression [73,74,75,76,77]. In a mouse model, overexpression of *LPAR2* induces intestinal dysplasia, which therefore alters the proliferation and differentiation of the intestinal epithelial cells (IEC) [78]. The pivotal inflammasome component, *Caspase-1*, is an emerging player in various diseases progressions, as well as a biomarker for early diagnosis and a therapeutic target [79,80,81,82]. The encoded protein of the *NHS* gene regulates the development of brain, craniofacial, eye, and tooth, in addition to actin remodeling and thus maintaining cell morphology. Mutations in the *NHS* gene were found to cause Nance–Horan syndrome (NHS), an X-linked disorder [83,84,85,86,87,88]. 

Ontology enrichment analysis of the genes provides insight into the role of the significantly methylated genes, showed that hypo- and hypermethylated CpG sites have occurred at various categories of biological processes, cellular components, and molecular functions. The most relevant genes class was associated with the Src-homology 2 (SH2) domain binding (GO:0042169). Interestingly, the genes in SH2 domain binding were hypermethylated (Table S1). Among them is the synaptophysin (*SYP*) gene, where SH2 domains bind to its multiple carboxyterminal tyrosine-phosphorylated residues [89]. SH2 domains are involved in various signaling transduction and found to regulate cellular behavior and functions upon interactions with other domains and cellular components [90]. However, the *SYP* gene is a pivotal regulator in the release of neurotransmitters and synaptic plasticity. It was found to be associated with some psychiatric disorders such as attention-deficit/hyperactivity disorder (ADHD) [91], schizophrenia [92], and depression [93]. Other prevailing ontological terms are the regulation of transcription, DNA-templated (GO:0006355) and histone exchange (GO:0043486). The latter of which process genes, acidic nuclear phosphoprotein 32 family member C and member E (*ANP32C* and *ANP32E,* respectively) and were also hypermethylated (Table S1). They were frequently found to regulate gene expression by acting on the substitution of histones or histone subunits within the chromatin, especially the *ANP32E* gene [94,95,96]. The *ANP32E* gene was enriched in two other cellular component terms, nucleus (GO:0005634) and Swr1 complex (GO:0000812). Although some ANP 32 family members function as tumor suppresser, *ANP32C* is an oncogene that has been overexpressed in prostate and breast cancer [97,98]. In a similar context, *ANP32E* is found to induce oncogenesis of triple-negative breast cancer (TNBC) by upregulation of the transcription factor, E2F1 [99]. Furthermore, those genes belong to the regulation of transcription, DNA-templated ontology, were represented by hypo- and hypermethylation patterns (Table S1). Among them is the ASXL transcriptional regulator 2 (*ASXL2*) gene, in which de novo germline truncation in ASXL2 variants have been associated with various clinical features of intellectual disabilities, macrocephaly, and dysmorphism [100]. Similarly, *ASXL2* deficiency in mice model (ASXL2−/−) was directed towards insulin resistance, osteopetrosis, and lipodystrophy [101]. Moreover, it promotes breast carcinogenesis through epigenetic regulation of the estrogen receptor alpha (ERα) [102].

## 5. Conclusions

In addition to the preliminary known genetic predisposition of keloids, methylation sequencing of CpG sites in keloids revealed significant hyper- and hypomethylated genes in fibroblasts from keloid scars compared to control fibroblasts demonstrated the vital role of DNA methylation as an epigenetic regulation mechanism in keloids formation and progression, as well as a putative therapeutic target, by reversing the methylation status to halt the overgrowth in scars. The DM genes and their products are known to elaborate several biological pathways, signaling, and functions that could be implicated further to understand keloid etiology and targeted treatment development in future exploration.

## Figures and Tables

**Table 1:** Patient and keloid scar details.

Subject ID ^1^	Age	Gender ^2^	Type of Tissue	Site of Tissue	Ethnicity
P 1	40	F	keloid scar	shoulder	East Asian
P 2	38	F	keloid scar	neck (thyroid)	Southeast European
P 3	29	M	keloid scar	forearm	Hispanic
P 4	30	M	keloid scar	sternum	Northwest European
P 5	53	M	keloid scar	sternum	Northwest European
P 6	28	M	keloid scar	upper arm	Northwest European
P 7	18	F	keloid scar	shoulder	East Asian
P 8	42	M	keloid scar	ear	Northwest European
P 9	30	M	keloid scar	sternum	East Asian
P 10	21	M	keloid scar	sternum	Northwest European and East Asian
P 11	47	F	keloid scar	sternum	Northwest European
P 12	29	M	keloid scar	sternum	East Asian
C 1	29	M	normotrophic scar	forearm	South East Asian
			normal skin	contralateral forearm	
C 2	25	M	normotrophic scar	forearm	Caucasian
			normal skin	contralateral forearm	
C 3	19	M	normotrophic scar	forearm	Caucasian
			normal skin	contralateral forearm	
C 4	25	M	normotrophic scar	forearm	Caucasian
			normal skin	contralateral forearm	
C 5	30	M	normotrophic scar	forearm	Caucasian
			normal skin	contralateral forearm	
C 6	19	M	normotrophic scar	forearm	Caucasian
			normal skin	contralateral forearm	

^1^ P: Patient; C: Control. ^2^ M: Male; F: Female.

**Table 2:** Gene ontology enrichment analyses of the genes associated with the top 100 CpG sites.

Category ^1^	Term	*p*-Value ^2^	Genes
MF	GO:0042169~SH2 domain binding	0.002	*SYP, NLK, DAG1*
BP	GO:0006355~regulation of transcription, DNA-templated	0.005	*ASXL2, PKNOX2, ZNF718, BPTF, TFEC, NLK, SCML1, GAS7, BRD8, KLF3*
CC	GO:0005634~nucleus	0.011	*ANP32C, NLK, ANP32E, SCML1, TKT, CAMKK1, TNKS2, PKNOX2, PSMA1, SERPINB9, ZNF718, FAM64A, SENP1, TAF15, BPTF, C19ORF66, USP36, FANCC, BRD8, MYH10, KLF3*
BP	GO:1903955~positive regulation of protein targeting to mitochondrion	0.016	*HSPA1L, NBPF3, USP36*
CC	GO:0005654~nucleoplasm	0.020	*ASXL2, NLK, FANK1, DAG1, TKT, HSPA1L, PSMA1, SENP1, BPTF, TAF15, TFEC, BRD8, FANCC*
CC	GO:0000812~Swr1 complex	0.021	*ANP32E, BRD8*
BP	GO:0043486~histone exchange	0.025	*ANP32C, ANP32E*
BP	GO:0071310~cellular response to organic substance	0.038	*SYP, CASP1*

^1^ BP: biological process; CC: cellular component; MF: molecular function; ^2^ Terms with a *p*-value ≤ 0.05 are shown.

## Supplementary Materials

The following are available online at https://www.mdpi.com/2227-9059/8/7/181/s1, Table S1: The top 100 CpG sites and the associated genes.
