# Supplementary material for: Identification of Differentially Methylated CpG Sites in Fibroblasts from Keloid Scars
Source: Biomedicines. 2020 Jun 28;8(7):181. doi: 10.3390/biomedicines8070181 (PMC7400180; doi:10.3390/biomedicines8070181)
Supplement: Supplementary file 1 [file biomedicines-08-00181-s001.pdf]

Table S1. The top 100 CpG sites and the associated genes.

CpG	Chr.[1]	Gene	Region	CpG Island	Mean β value (CF)[2]	Mean β value (KF)[2]	Mean β value diff (KF-CF)[2]	mean.quot. (log2)[3]	FDR[4]	Combined rank score	Methylation pattern
cg11963436	chr10	TNKS2	Body	NA	0.0655	0.5151	0.4496	2.7984	2.32E-10	180	Hypermethylation
cg04294190	chrX	FAM45B	Body	NA	0.0602	0.4418	0.3816	2.6854	8.20E-09	325	Hypermethylation
cg05396017	chrX	NA	NA	N_Shore	0.1169	0.7131	0.5962	2.5103	5.54E-10	464	Hypermethylation
cg12050434	chr12	NA	NA	NA	0.1124	0.5722	0.4597	2.2494	2.32E-10	912	Hypermethylation
cg21581312	chr15	LOC723972	TSS200	NA	0.1569	0.7604	0.6035	2.2067	7.98E-10	1014	Hypermethylation
cg24958366	chr17	NA	NA	NA	0.1492	0.6536	0.5044	2.0594	2.35E-10	1352	Hypermethylation
cg25365518	chr17	GAS7	Body	NA	0.1178	0.4692	0.3514	1.9064	1.58E-08	1775	Hypermethylation
cg08700284	chr7	RHBDD2	TSS200	Island	0.1058	0.4053	0.2995	1.8426	2.28E-09	1992	Hypermethylation
cg04657325	chr4	NA	NA	NA	0.4013	0.1127	-0.2887	-1.7456	6.10E-08	2378	Hypomethylation
cg24400517	chr7	LOC100134229;JHDM1D	TSS1500;Body	Island	0.0945	0.3725	0.2780	1.8718	2.23E-07	2506	Hypermethylation
cg21285133	chr1	ANP32E	TSS1500	S_Shore	0.1292	0.4416	0.3124	1.6976	6.09E-08	2558	Hypermethylation
cg19713980	chr17	CAMKK1	5'UTR	Island	0.0396	0.3163	0.2766	2.7164	1.83E-07	2616	Hypermethylation
cg00555695	chr8	PVT1	Body	NA	0.1373	0.4601	0.3228	1.6746	2.31E-07	2645	Hypermethylation
cg00429618	chr9	ZNF322B	TSS1500	Island	0.0943	0.3699	0.2756	1.8654	2.74E-07	2680	Hypermethylation
cg01852095	chr2	LRP1B	Body	NA	0.2005	0.6565	0.4560	1.6630	1.97E-08	2695	Hypermethylation
cg00563768	chr17	TAF15	3'UTR	NA	0.2326	0.7486	0.5160	1.6449	1.17E-08	2793	Hypermethylation
cg25444615	chr19	NA	NA	NA	0.1381	0.4411	0.3030	1.6067	6.96E-08	2986	Hypermethylation
cg01262337	chr3	TKT	TSS200	Island	0.1459	0.4642	0.3183	1.6052	3.09E-07	2995	Hypermethylation
cg24449629	chr19	NA	NA	S_Shelf	0.0646	0.3355	0.2709	2.2123	1.68E-07	3024	Hypermethylation
cg21152376	chr3	NA	NA	Island	0.3971	0.1245	-0.2726	-1.5978	5.18E-10	3035	Hypomethylation

Table S1. Continued.

CpG	Chr.[1]	Gene	Region	CpG Island	Mean β value (CF)[2]	Mean β value (KF)[2]	Mean β value diff (KF-CF)[2]	mean.quot. (log2)[3]	FDR[4]	Combined rank score	Methylation pattern
cg17824939	chr12	C12orf50	5'UTR	NA	0.2113	0.6515	0.4401	1.5794	1.77E-07	3114	Hypermethylation
cg09234578	chrX	NHS	Body	Island	0.0607	0.3282	0.2675	2.2580	2.40E-08	3332	Hypermethylation
cg01082910	chr10	NA	NA	N_Shore	0.1774	0.5335	0.3561	1.5358	3.16E-07	3365	Hypermethylation
cg17461271	chr1	NA	NA	NA	0.2997	0.8836	0.5839	1.5287	1.42E-10	3409	Hypermethylation
cg05899741	chr4	NA	NA	NA	0.1434	0.4298	0.2863	1.5191	9.81E-09	3471	Hypermethylation
cg27248073	chr17	FAM64A	TSS200	Island	0.4570	0.1573	-0.2997	-1.4809	2.30E-08	3754	Hypomethylation
cg11450715	chr2	NA	NA	Island	0.5710	0.2020	-0.3690	-1.4545	3.96E-09	3921	Hypomethylation
cg16900255	chr15	C15orf59	Body	NA	0.2710	0.7519	0.4809	1.4390	2.83E-08	4053	Hypermethylation
cg24132580	chr4	MARCH1;ANP32C	5'UTR;TSS200	NA	0.0661	0.3252	0.2591	2.1388	1.54E-07	4205	Hypermethylation
cg07441824	chrX	ZCCHC16	5'UTR	Island	0.2428	0.6654	0.4225	1.4175	8.75E-08	4226	Hypermethylation
cg11792281	chr17	NLK	Body	NA	0.3148	0.8473	0.5326	1.4005	3.27E-08	4383	Hypermethylation
cg10044516	chrX	NA	NA	Island	0.2873	0.7638	0.4765	1.3799	5.14E-09	4590	Hypermethylation
cg10818284	chrX	SYP	Body	Island	0.0550	0.4606	0.4056	2.8559	6.52E-07	4879	Hypermethylation
cg02449698	chr1	EFCAB7;DLEU2L	Body;TSS200	NA	0.3210	0.8321	0.5111	1.3472	1.42E-10	4940	Hypermethylation
cg16752652	chr5	SLC12A2;FLJ33630	1stExon;TSS1500	Island	0.1014	0.3526	0.2513	1.7030	1.09E-09	5168	Hypermethylation
cg14018420	chr1	NBPF3	TSS1500	Island	0.6898	0.2696	-0.4201	-1.3233	2.99E-09	5193	Hypomethylation
cg19541697	chr2	ASXL2	Body	NA	0.2586	0.6534	0.3948	1.3046	9.43E-08	5396	Hypermethylation
cg14502625	chr5	NA	NA	NA	0.2293	0.5852	0.3559	1.3146	7.83E-07	5524	Hypermethylation
cg18803079	chr1	EFCAB7;DLEU2L	Body;TSS200	NA	0.3768	0.9332	0.5564	1.2859	6.38E-14	5653	Hypermethylation
cg22805491	chr14	NA	NA	NA	0.3643	0.9019	0.5376	1.2846	1.85E-10	5673	Hypermethylation
cg20673919	chr11	PATE4	TSS1500	NA	0.1926	0.5101	0.3175	1.3603	8.15E-07	5735	Hypermethylation

Table S1. Continued.

CpG	Chr.[1]	Gene	Region	CpG Island	Mean β value (CF)[2]	Mean β value (KF)[2]	Mean β value diff (KF-CF)[2]	mean.quot. (log2)[3]	FDR[4]	Combined rank score	Methylation pattern
cg19933320	chr7	ZNF107	TSS1500	N_Shore	0.2004	0.4996	0.2992	1.2764	5.44E-07	5788	Hypermethylation
cg24568548	chr11	NA	NA	S_Shore	0.3454	0.8471	0.5017	1.2700	3.51E-09	5878	Hypermethylation
cg16435601	chr17	BPTF	1stExon	S_Shore	0.5014	0.2028	-0.2986	-1.2649	1.27E-07	5936	Hypomethylation
cg22336806	chr6	SERPINB9	TSS200	Island	0.0462	0.2922	0.2460	2.4256	8.28E-07	5952	Hypermethylation
cg01426921	chr9	RMRP;CCDC107	TSS1500;Body	Island	0.1968	0.4865	0.2897	1.2634	2.47E-08	5958	Hypermethylation
cg07707039	chr17	COX11	Body	N_Shelf	0.2431	0.5943	0.3512	1.2553	4.95E-07	6071	Hypermethylation
cg05001044	chr1	MIR1977	TSS1500	NA	0.3810	0.1374	-0.2436	-1.4075	5.48E-09	6310	Hypomethylation
cg14559409	chr10	NA	NA	Island	0.2950	0.7010	0.4060	1.2211	1.78E-07	6601	Hypermethylation
cg25034991	chr10	NA	NA	NA	0.1703	0.7647	0.5944	2.1036	9.72E-07	6622	Hypermethylation
cg13116816	chr7	C7orf23	Body	NA	0.2398	0.5690	0.3291	1.2126	4.40E-07	6749	Hypermethylation
cg10342490	chr11	GUCY1A2	Body	NA	0.3153	0.7430	0.4277	1.2108	3.02E-09	6785	Hypermethylation
cg07967114	chr12	NA	NA	NA	0.3250	0.7642	0.4392	1.2086	5.29E-09	6829	Hypermethylation
cg01070250	chr1	NA	NA	NA	0.3610	0.1213	-0.2397	-1.4987	1.44E-09	6954	Hypomethylation
cg27556136	chrX	NCRNA00182	Body	Island	0.3770	0.8789	0.5019	1.1998	3.15E-10	6987	Hypermethylation
cg05337192	chr1	NA	NA	NA	0.2140	0.5020	0.2880	1.1924	4.81E-08	7122	Hypermethylation
cg22281935	chr2	NA	NA	S_Shelf	0.1861	0.4344	0.2483	1.1802	1.09E-06	7362	Hypermethylation
cg09048334	chr6	NA	NA	Island	0.1428	0.3780	0.2352	1.3444	1.47E-09	7754	Hypermethylation
cg27069616	chr7	NA	NA	NA	0.2762	0.6266	0.3504	1.1535	1.06E-06	7865	Hypermethylation
cg06493963	chr7	TFEC	Body	NA	0.2841	0.6435	0.3593	1.1516	8.81E-07	7897	Hypermethylation
cg00090782	chr10	NA	NA	Island	0.4192	0.1580	-0.2612	-1.3536	1.23E-06	7908	Hypomethylation
cg27212541	chr3	DAG1	TSS200	Island	0.1711	0.5155	0.3444	1.5366	1.24E-06	7990	Hypermethylation

Table S1. Continued.

CpG	Chr.[1]	Gene	Region	CpG Island	Mean β value (CF)[2]	Mean β value (KF)[2]	Mean β value diff (KF-CF)[2]	mean.quot. (log2)[3]	FDR[4]	Combined rank score	Methylation pattern
cg12739584	chr6	NA	NA	N_Shore	0.0444	0.5345	0.4902	3.3243	1.29E-06	8185	Hypermethylation
cg01385356	chr17	USP36	TSS1500	Island	0.1839	0.4167	0.2328	1.1377	3.68E-08	8214	Hypermethylation
cg19463471	chr19	C19orf66	Body	Island	0.1117	0.3441	0.2324	1.5409	1.13E-06	8244	Hypermethylation
cg12977827	chr5	BRD8	Body	N_Shelf	0.2339	0.5255	0.2916	1.1346	8.29E-07	8288	Hypermethylation
cg14115740	chr9	FANCC	5'UTR	Island	0.3679	0.8156	0.4477	1.1275	2.06E-08	8459	Hypermethylation
cg05294909	chrX	SCML1	5'UTR	Island	0.0781	0.3089	0.2309	1.8567	1.04E-07	8547	Hypermethylation
cg22502448	chr3	ZNF167	Body	Island	0.3059	0.6776	0.3717	1.1221	8.19E-07	8585	Hypermethylation
cg05893889	chr2	NA	NA	NA	0.3429	0.7539	0.4109	1.1139	2.01E-09	8745	Hypermethylation
cg13876481	chr6	C6orf103;LOC729176	Body;Body	NA	0.1258	0.3551	0.2293	1.4270	1.25E-07	8845	Hypermethylation
cg14188774	chr7	NA	NA	NA	0.0717	0.3282	0.2565	2.0495	1.47E-06	8976	Hypermethylation
cg01381374	chr7	NA	NA	NA	0.3198	0.6969	0.3771	1.1000	1.44E-07	9060	Hypermethylation
cg18700146	chr3	GRAMD1C	Body	N_Shore	0.3279	0.7108	0.3829	1.0929	7.03E-09	9227	Hypermethylation
cg03243683	chr6	HSPA1L	Body	N_Shelf	0.3680	0.7957	0.4277	1.0919	1.32E-09	9248	Hypermethylation
cg18458431	chr19	LPAR2	TSS200	Island	0.0800	0.3064	0.2264	1.8137	4.00E-08	9446	Hypermethylation
cg24159721	chr1	NA	NA	NA	0.3646	0.1384	-0.2262	-1.3359	9.44E-09	9491	Hypomethylation
cg13802966	chr11	CASP1	Body	NA	0.4001	0.1742	-0.2259	-1.1549	1.86E-08	9542	Hypomethylation
cg26829098	chr1	NA	NA	NA	0.2105	0.4554	0.2449	1.0776	1.30E-06	9623	Hypermethylation
cg25724260	chr7	NA	NA	NA	0.0839	0.3384	0.2545	1.8910	1.64E-06	9677	Hypermethylation
cg14394011	chr17	MYH10	Body	NA	0.3135	0.6719	0.3584	1.0757	1.26E-07	9690	Hypermethylation
cg26868250	chr11	PSMA1	Body	NA	0.2845	0.6100	0.3254	1.0737	1.29E-08	9738	Hypermethylation
cg05062854	chr22	NA	NA	N_Shelf	0.2440	0.5240	0.2801	1.0723	2.30E-08	9778	Hypermethylation

Table S1. Continued.

CpG	Chr.[1]	Gene	Region	CpG Island	Mean β value (CF)[2]	Mean β value (KF)[2]	Mean β value diff (KF-CF)[2]	mean.quot. (log2)[3]	FDR[4]	Combined rank score	Methylation pattern
cg08472559	chr10	FANK1	TSS200	Island	0.3696	0.1453	-0.2244	-1.2897	1.89E-08	9863	Hypomethylation
cg08941173	chr11	PKNOX2	5'UTR	Island	0.3994	0.1755	-0.2240	-1.1423	1.70E-08	9947	Hypomethylation
cg23596249	chr4	MARCH1;	5'UTR	Island	0.2972	0.6322	0.3351	1.0641	1.41E-07	10002	Hypermethylation
cg05041728	chrX	ZCCHC16	5'UTR	Island	0.4125	0.8724	0.4599	1.0625	2.19E-09	10058	Hypermethylation
cg21036194	chr5	SNCAIP	Body	NA	0.4592	0.9687	0.5095	1.0605	1.40E-06	10110	Hypermethylation
cg24776295	chrX	NA	NA	N_Shore	0.2850	0.6046	0.3196	1.0590	1.53E-07	10142	Hypermethylation
cg24232869	chr12	SENP1	Body	NA	0.2656	0.5630	0.2974	1.0561	4.99E-07	10211	Hypermethylation
cg15792688	chr4	ZNF718	Body	Island	0.4204	0.1979	-0.2225	-1.0500	3.79E-07	10382	Hypomethylation
cg23113715	chr22	NA	NA	Island	0.4407	0.2079	-0.2328	-1.0486	9.01E-08	10414	Hypomethylation
cg15732422	chr12	CASC1	Body	N_Shelf	0.3092	0.6498	0.3406	1.0475	4.67E-09	10458	Hypermethylation
cg07158881	chr17	NA	NA	N_Shelf	0.3474	0.1258	-0.2216	-1.3958	4.06E-07	10491	Hypomethylation
cg14094333	chr4	KLF3;KLF3;FLJ13197	1stExon;5'UTR;Body	Island	0.2901	0.0687	-0.2215	-1.9315	1.85E-10	10520	Hypomethylation
cg14222140	chr1	C1orf201	Body	NA	0.2863	0.6006	0.3143	1.0433	3.16E-07	10580	Hypermethylation
cg00645010	chr1	MIR1977	TSS1500	NA	0.2678	0.0467	-0.2211	-2.2918	2.99E-09	10601	Hypomethylation
cg26771998	chr10	BMS1P4	TSS200	Island	0.3560	0.1353	-0.2207	-1.3326	4.69E-09	10690	Hypomethylation
cg24376286	chr2	NA	NA	NA	0.3199	0.6654	0.3454	1.0334	3.58E-09	10873	Hypermethylation
cg00609367	chr10	NA	NA	NA	0.2464	0.5126	0.2662	1.0275	1.72E-06	11038	Hypermethylation

[1] Chr: chromosome.
[2] Mean β value: mean methylation levels across all sites in a region; CF: control fibroblast; KF: keloid fibroblast (KF).
[3] mean.mean.quot.log2: mean quotient in means across all sites in a region.
[4] FDR: False discovery rate.
